# Case of Chronic Dysentery Cured by Large Doses of Ipecacuana

**Published:** 1881-06-20

**Authors:** W. S. Whitwell


### CASE OF CHRONIC DYSENTERY CURED BY LARGE DOSE OF IPECACUANA.

BY W. S. WHITWELL, M. D.

Geo. L., mulatto, aged twenty-one, born in Jamaica. At the age of ten years went to Peru. When seventeen years old he had an attack of dysentery at Calloa. Passed blood with each stool, and at times had between twenty and thirty a day. After seven months he apparently recovered without treatment. Four months later he came to San Francisco, and on coming into the harbor had an attack of diarrhœa, which lasted ten days. When in Virginia City he was obliged to enter the hospital on account of still another attack which was soon checked, but which returned on his going to work. During the following year had three attacks of dysentery. In the fall of 1878, while in San Francisco, in consequence of a fresh attack he went to several of the hospitals in his endeavor to be cured; says that he took many kinds of medicine, and was at one time placed for a month or six weeks on milk diet, but still was not relieved. In September, 1879, he was brought to me looking pale, thin and enfeebled. Said that he had no natural passage, but merely a slimy, watery discharge, which was mixed with blood; that he felt much griping and was forced to go to the closet nearly every twenty minutes; that at night he was up from six to a dozen times; that he never slept soundly except for a few minutes, and that he was constantly grinding his teeth.

Told patient to go home and go to bed; to place a mustard poultice on the epigastric region, and to take one-third of the following prescription:

℞ **Pulv. Ipecac** ........................................ ʒ ijss.

**Mucil. Acaciæ** ........................................ ℥ ij.

Patient, through a misunderstanding, took the whole, but kept it on his stomach with the aid of the mustard for three-quarters of an hour. He then thinks that he vomited about one-half. Had one passage about two hours later. Since this time he has had no griping, has slept soundly, and has had but one passage every day. There was even a tendency towards constipation. In October, one month after treatment, I saw him again; he had gained in flesh and strength. His stools were dark colored and natural, such as he had not had for more than a year. His diet, although he had restricted it at first, was now ample and varied. In January he had gained still more, and was looking well and worked regularly. About a year afterwards, having a slight diarrhœa, and fearing a return of his old trouble, he had the medicine renewed and repeated the dose of two drachms and a half with good effect.

This case has been reported for the purpose of showing that it is possible to give large doses of ipecac without injury, and that properly administered it is not necessary to combine it with opium, as is commonly recommended. As will be seen in an article on acute dysentery, among the selections, the largest dose given by any one is that by Bateman of two drachms, but he deems it necessary to give with it one drachm of opium, presumedly to control the vomiting. In the above case the vomiting was not severe, not more so than that attendant on much smaller doses. We cannot consider the vomiting as beneficial, especially when a patient is much weakened, and think that the best way to avoid it is to give a sufficiently large dose the first time.

The second day out, on leaving Hong-Rong for San Francisco, in 1879, a sea captain six feet in height and weighing about one hundred pounds, came into my room and said that I must do something for him and that quickly. He had had dysentery before reaching Hong-Kong; had been given medicine which checked the disease, and now, two days later, it had come on again. As he told me afterwards, well knowing its dangerous character in the tropics, he never expected to reach San Francisco. Ipecac and a milk diet cured him, but the drachm dose had to be repeated, weak as he was, before that result was attained. The conclusion drawn is that a larger dose would have saved repetition and added to the comfort of the patient. That he was cured, however, I am assured, for when he came into my office six months afterwards he weighed one hundred and eighty pounds.—*Western Lancet.*

